# Fault Diagnosis of Motor Bearing Transmission System Based on Acoustic Characteristics

**DOI:** 10.3390/s26010259

**Published:** 2025-12-31

**Authors:** Long Ma, Yan Zhang, Zhongqiu Wang

**Affiliations:** 1 China Coal Technology & Engineering Group (CCTEG) Shenyang Research Institute, Shenyang 113122, China; malongccteg@163.com (L.M.); zhangyanccteg@sina.com (Y.Z.); 2 School of Mechanical and Electrical Engineering, China University of Mining and Technology, Xuzhou 221116, China

**Keywords:** Mel frequency cepstral coefficient, deep learning, fault diagnosis, LSTM, acoustic features, attention mechanism

## Abstract

Traditional vibration-based methods for bearing fault diagnosis, while prevalent, often require contact measurement, and sound signal is a broadband signal relative to the vibration signal. To overcome these limitations, this paper explores the advantages of acoustic signals, non-contact sensing, and rich broadband information and proposes a fault diagnosis framework based on acoustic features and deep learning. The core of our method is a CNN–attention mechanism–LSTM model, specifically designed to process one-dimensional sequential features: the 1D-CNN extracts local features from Mel frequency cepstral coefficient (MFCC) features, the attention mechanism (selecting ECA as the optimal solution) selectively enhances features, and the LSTM captures temporal dependencies, collectively enabling effective classification of fault types. Furthermore, to enhance model efficiency, a ReliefF-based feature selection algorithm is employed to identify and retain only the most discriminative acoustic features. Experimental results demonstrate that the proposed method achieves an average diagnostic accuracy of 99.90% in distinguishing normal, inner-ring, outer-ring, and mixed-defect bearings. Notably, results show that after using the feature selection algorithm, the number of parameters and the estimated total size are significantly reduced while ensuring that the accuracy remains basically unchanged. This work validates the effectiveness of non-contact solutions for bearing fault diagnosis using acoustic features and has enormous potential for industrial applications.

## 1. Introduction

The transmission system is a mechanical system that comprises gears, bearings, and other mechanical components, essential for transmitting power or motion from one point to another [1]. Bearings, a prevalent mechanical component utilized across diverse fields and industries, play a crucial role in this system [2]. In the context of the country’s advancing development, there exists a substantial demand for bearings as indispensable consumables in the manufacturing process. Bearings serve functions such as friction reduction, load support, and precise positioning provision [3]. Thus, the significance of diagnosing bearing faults is inherently clear.

Currently, the mainstream approach for diagnosing bearing faults involves utilizing vibration signals [4,5,6,7]. Various methods are employed for this purpose, such as Wigner–Ville [8], spectrum auto-correlation analysis [9], sparsogram [10], KS test [11], wavelet transform [12], envelope analysis [13], wavelet denoising [14], cepstrum calculation [15], empirical mode decomposition [16], morphological wavelet slices [17], tachometer-less synchronously averaged envelope feature extraction technique [18], and envelope order tracking analysis [19], among others. Despite the prevalence of these methods, the utilization of wider bandwidth sound signals for bearing fault diagnosis remains relatively uncommon in comparison with traditional narrowband vibration signals. However, certain researchers have begun exploring this avenue [20,21]. Common situations for using sound signals aim to process transformer fault problems. Common methods include data-driven [22], poly-phase filtering and complex variational modal decomposition [23], Mel-GADF and ConvNeXt-T networks [24], support vector machine [25], WKPCA-WM and IPOA-CNN networks [26], Mel-spectrum convolutional neural network [27], blind source separation [28], improved MFCC and 3D-CNN networks [29], multi-characteristic voiceprint maps [30], etc.

The vibration signal is a narrowband signal, that is, the frequency band of the signal is narrow and the main frequency components of the signal are concentrated within a certain frequency range. The quality of sound signals is often determined by the frequency range of the sound. Therefore, sound signals are considered to be broadband signals. The frequency range of broadband signals is wider, and its main frequency components are distributed in a very wide frequency range [31]. Compared with traditional vibration signals, sound signals have the advantage of a wide frequency band. In some practical situations, vibration sensors may not be suitable for contact measurement due to harsh conditions. However, acoustic sensors offer the advantage of non-contact data collection by capturing sound signals, which is also one of the advantages [21].

Existing studies on bearing fault diagnosis can be broadly categorized by their learning paradigms. Supervised learning approaches require fully labeled datasets to train models for direct classification or regression. This category encompasses a wide range of methods, from traditional classifiers like support vector machines (SVMs) to modern deep neural networks such as convolutional neural networks (CNNs) and long short-term memory (LSTM) networks, which have been mainstream in vibration-based diagnosis [4,16,20]. Notably, advanced frameworks like the generalized Koopman neural operator demonstrate how deep learning can be integrated with dynamical system theory for efficient and high-fidelity modeling of complex mechanical systems [32]. In contrast, semi-supervised or self-supervised approaches have gained prominence for scenarios with limited labeled data, a common challenge known as data scarcity. These methods leverage both labeled and unlabeled samples to improve model generalization. A representative state-of-the-art method in this category is the adaptive fused domain-cycling variational generative adversarial network (AFDVGAN), which synthesizes high-quality data to augment scarce real samples, enabling robust fault diagnosis under imbalance conditions [33].

**Figure 1:**
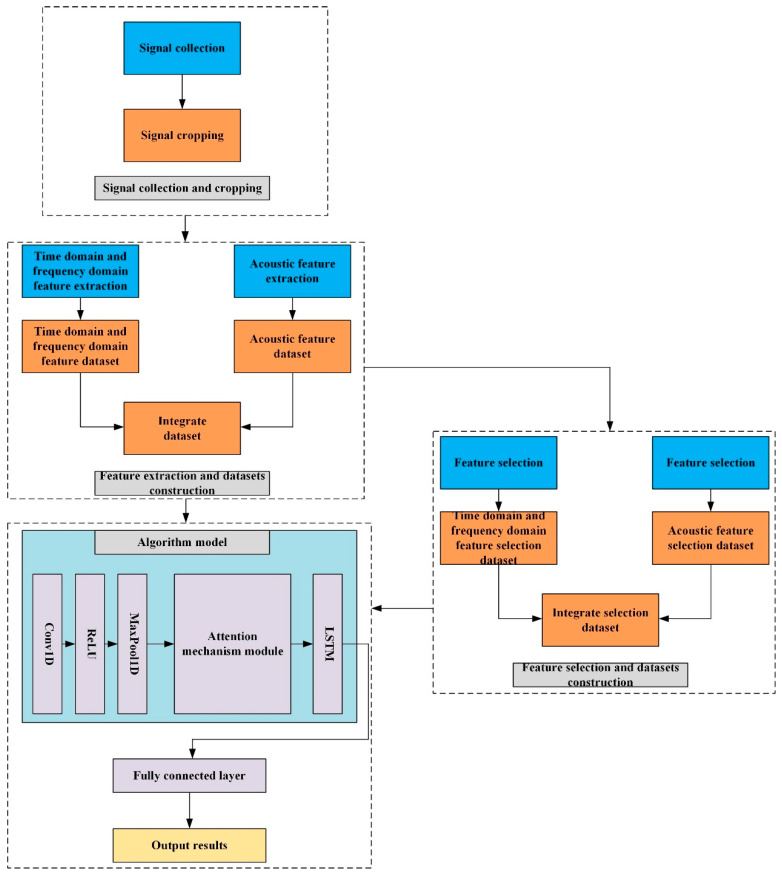
Process of the fault diagnosis of motor bearing transmission system.

However, directly applying acoustic methods developed for transformers to bearing fault diagnosis faces specific challenges. These include the strong background noise in mechanical environments and the need for efficient model architectures suitable for one-dimensional feature inputs. To address these challenges and leverage the non-contact broadband advantages of acoustic signals, this paper proposes a novel fault diagnosis framework for bearing fault diagnosis. The overall process of this paper is shown in Figure 1. The main contributions of this work are summarized as follows:

(1) This study uses the unique acoustic feature of the Mel frequency cepstral coefficient (MFCC) of acoustic signals to provide a feasible non-contact bearing fault diagnosis scheme.
(2) We design a neural network architecture that integrates 1D-CNN for local feature ex-traction, an attention mechanism (with ECA selected as optimal) for highlighting critical information, and LSTM for capturing temporal dependencies. This model is specifically tailored for processing the sequential one-dimensional acoustic features.
(3) By applying the ReliefF algorithm, we successfully reduce the acoustic feature dimension from 39 to 18. The results show that after using the feature selection algorithm, the number of parameters and the estimated total size are significantly reduced while ensuring that the accuracy remains basically unchanged, proving the feasibility of using a minimal yet effective feature set.

The rest of this paper is organized as follows: Section 2 presents the methodology and principles. Section 3 gives detailed information on the experimental setup and dataset. Section 4 describes the proposed algorithm and analyzes the training results. Finally, Section 5 draws the conclusion.

## 2. Methodology and Principles

The core modules function as follows:

1. Acoustic feature extraction module: Extracts the unique acoustic features of the sound signal, Mel frequency cepstral coefficients (MFCCs), as inputs to the network model.
2. CNN–attention mechanism–LSTM model:
  (1) One-dimensional convolutional layers: Automatically learn and extract locally sensitive patterns and abstract representations from the input feature sequences.
  (2) Attention mechanism: Dynamically calibrates the importance weights of different feature channels, enhancing the model’s ability to focus on critical discriminative information.
  (3) Long short-term memory (LSTM) network: Captures long-term dependencies and dynamic evolution patterns within the feature time series.
3. Feature selection module: Applies the ReliefF algorithm to filter out feature subsets with higher weights, reduce data dimensionality, and improve model training efficiency.

### 2.1. Feature Extraction

First, we need to perform feature extraction operations to extract one-dimensional features as input for the task.

Scholars typically extract the time domain features and frequency domain features of the bearing [4,34,35,36,37]. In this paper, we extracted a total of 22 time domain features and frequency domain features. The names and abbreviations of the extracted features can be found in Table 1.

**Figure 2:**
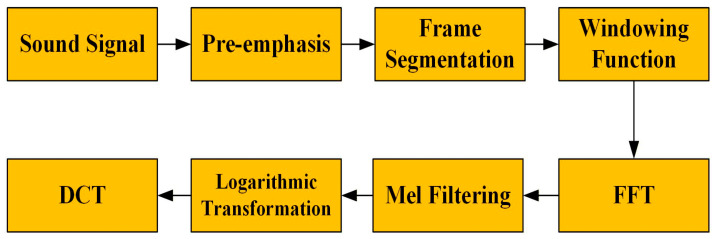
Mel frequency cepstral coefficient extraction steps.

Compared with vibration signals, sound signals also have the capability to extract their acoustic features. The Mel frequency cepstral coefficient (MFCC) is a feature widely used in audio processing and speech recognition [38,39]. In this study, we have opted to extract the Mel frequency cepstral coefficients (MFCCs). The steps involved in extracting this feature are illustrated in Figure 2.

1. Pre-emphasis: First, the signal needs to be pre-emphasized to enhance the high frequency part. Pre-emphasis can help balance the spectrum and prevent numerical problems.
2. Frame segmentation: Divide the continuous sound signal into short frames.
3. Windowing: Apply a window function to each frame, which is usually a Hamming window or a Hanning window.
4. Fast Fourier transform (FFT): The frame signal after the window function is applied is transformed by fast Fourier transform to obtain the spectrum.
5. Mel filter filtering: The spectrum is filtered through a set of Mel filter banks to simulate the perception behavior of the human ear.
6. Logarithmic transformation: Take the logarithm of the energy of the Mel filter bank. This is because human hearing perceives the amplitude and frequency of audio in a logarithmic way, so this transformation makes the data closer to human perception; on the other hand, the logarithm can also compress the range of the data and overcome the impact of large values.
7. Discrete cosine transform (DCT): The energy of the Mel filter bank after logarithm is subjected to discrete cosine transform. The purpose of this is mainly to remove the correlation between the filter energy coefficients and retain the most important cepstral coefficients.

After obtaining the MFCC features corresponding to the sound signal through the aforementioned steps, it is important to note that the basic MFCC features primarily capture static characteristics. In order to further enhance the comprehensiveness of the acoustic feature dataset, additional dynamic features are extracted, including the first-order differential MFCC and the second-order differential MFCC [22]. Each of these 3 feature sets encompasses 13 distinct features, thereby resulting in a total of 39 features within the entire acoustic feature dataset.

### 2.2. Convolutional Neural Network Theory

Convolutional neural networks, known as CNNs, are multi-layer neural network models that typically include convolutional layers, pooling layers, and activation functions. Primarily designed for processing image data, CNNs can also be applied to analyze video, text, sound data, or one-dimensional features [40,41]. Due to the strong fitting capabilities inherent in neural networks, CNNs are capable of extracting both traditional topological structure features and high-dimensional abstract features that may be challenging to represent using mathematical models [21].

The one-dimensional convolutional neural network is a variant of the widely known convolutional neural network. Specifically designed for handling sequence data like time series, signal data, or text data, it has proven to be more efficient and practical for processing such types of information. In contrast to the two-dimensional convolutional neural network, which is predominantly used in image processing applications, the one-dimensional convolutional neural network stands out for its effectiveness in processing sequential datasets [42].

The convolution layer is the core of the convolutional neural network. It uses the convolution kernel matrix to perform local convolution operations to extract the implicit correlation in the input data to achieve feature extraction.

The activation function is used to introduce nonlinear factors, allowing the network to learn complex patterns. Compared with the Sigmoid function and the Tanh function, the ReLU activation function has fast convergence and better model generalization ability.

The expression of the ReLU function is as follows:

(1) f(x)=max(0,x).

where x is the input and f(x) is the output.

The feature mapping layer, also known as the pooling layer, plays a crucial role in deep learning models. It is designed to reduce the dimensions of features while preserving essential information and improving the model’s generalization capabilities by minimizing the number of parameters and computational load. Among the various pooling operations available, such as average pooling, the maximum pooling operation is chosen for this study.

After the convolutional layer and the pooling layer, a convolutional neural network typically consists of one or more fully connected layers. The fully connected layer is responsible for integrating high-level features, mapping them to the output space, and adjusting the network output.

Based on these characteristics, CNNs have been extensively applied in image analysis, face recognition, speech recognition, and various other fields [40,41,42].

### 2.3. Attention Mechanism Theory

While CNN extracts informative hierarchical features, not all learned features contribute equally to fault discrimination. Therefore, we use the attention mechanism module. This technique enhances the model’s capability to focus on critical segments of the feature sequence, thereby improving its overall performance.

The attention mechanism, a technique utilized in deep learning, enhances the model’s capability to concentrate on specific pieces of information by mirroring the operational mechanism of human attention. This technique enables the model to prioritize crucial segments when handling extensive data, thereby improving its focus, interpretability, and performance within a sequence model while facilitating information integration. Through the integration of the attention mechanism, neural networks gain the ability to autonomously learn and selectively emphasize key details from the input data, consequently augmenting the model’s overall performance and generalization capacity [43].

### 2.4. Long Short-Term Memory Network Theory

Long short-term memory (LSTM) is a specialized form of recurrent neural network (RNN) utilized for handling and forecasting long-term dependencies in sequential data. Unlike traditional RNNs, LSTM is engineered to address challenges such as gradient vanishing and exploding that emerge when processing lengthy sequence data. By incorporating the gating mechanism, LSTM retains information via the cell state, where the forget gate is responsible for dictating the removal of irrelevant information, the input gate governs the addition of new information to the cell state, and the output gate influences the value output based on the existing cell state [44].

The specific calculation formula in LSTM is shown as follows:

Forget gate:

(2) $$f_{t}=\phi (W_{f}[h^{\left(t-1\right)},x^{\left(t\right)}]+b_{f})$$

where $h^{\left(t-1\right)}$ is the hidden state at the previous moment, and $x^{\left(t\right)}$ is the input at the current moment.

Input gate:

(3) $$i_{t}=\phi (W_{i}[h^{\left(t-1\right)},x^{\left(t\right)}]+b_{i})$$

(4) $$\tilde{c^{\left(t\right)}}=\tanh(W_{c}[h^{\left(t-1\right)},x^{\left(t\right)}]+b_{c})$$

(5) $$c^{\left(t\right)}=f_{t}⊙c^{\left(t-1\right)}+i_{t}⊙\tilde{c^{\left(t\right)}}$$

where $c^{\left(t\right)}$ is the cell state at the current moment, and $\tilde{c^{\left(t\right)}}$ is the candidate vector.

Output gate:

(6) $$o_{t}=\phi (W_{o}[h^{\left(t-1\right)},x^{\left(t\right)}]+b_{o})$$

(7) $$h^{\left(t\right)}=o_{t}⊙\tanh(c^{\left(t\right)})$$

Due to its ability to handle long-term dependencies, LSTM has become an important tool for processing time series data.

### 2.5. Loss Function Theory

The loss function is a function used to quantify the difference between the predicted value and the true value of a model. It is a scalar that measures the “degree of error” of a model.

The cross-entropy loss function is the most crucial loss function in classification tasks, and its mathematical derivation is based on information entropy theory. It guides model optimization by measuring the difference between predicted probability distributions and true distributions.

The cross-entropy loss function avoids the gradient vanishing problem in the Sigmoid and Softmax saturation region, providing a stable and efficient update direction for optimizers.

## 3. Experimental Setup and Dataset Construction

### 3.1. Experimental Setup

**Figure 3:**
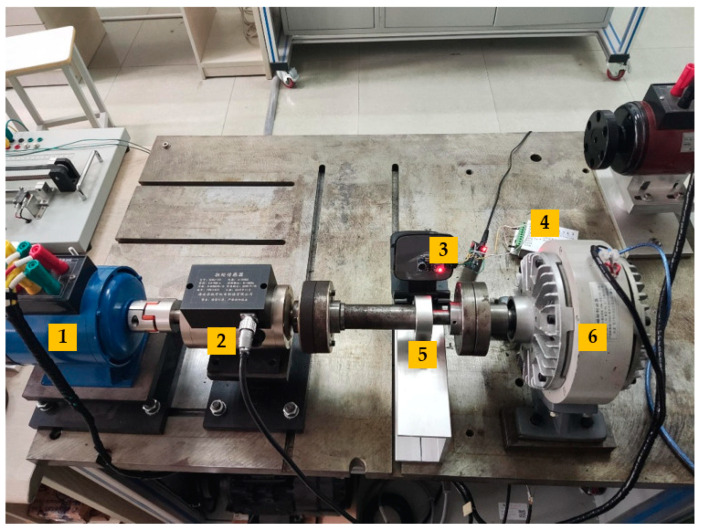
The entire experimental platform.

**Figure 4:**
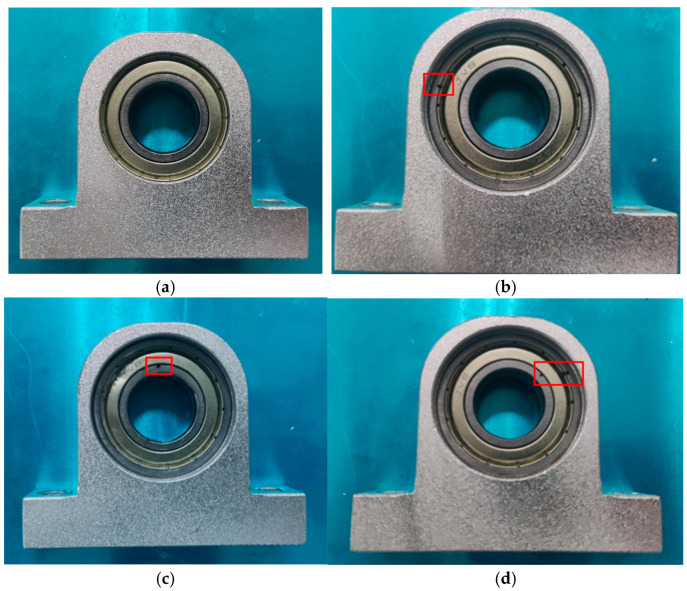
Bearings from left to right are (**a**) the normal intact state bearing, (**b**) the bearing of outer ring defect, (**c**) the bearing of inner ring defect, and (**d**) the bearing of mixed inner and outer defects.

The experimental platform described in this paper comprises several key components, including a magnetic powder brake, torque sensor, three-phase asynchronous motor, rolling bearing, acoustic sensor, data acquisition card, bearing seat, and bracket. Specifically, the setup features an HKDJ-26 three-phase asynchronous motor, an HCNJ-101 torque sensor, a CZ-2 magnetic powder brake, a DAQ122 data acquisition card, an LM386 acoustic sensor, and P6004 closed bearings with an inner diameter of 20 mm. Additionally, 0.5 mm hole defects have been intentionally created on both the inner and outer rings of the bearing, resulting in three types of defects: inner ring defect, outer ring defect, and mixed inner and outer defects. Figure 3 illustrates the layout of the entire setup. In the figure, the components are labeled as follows: 1 represents the three-phase asynchronous motor, 2 stands for the torque sensor, 3 represents the acoustic sensor, 4 represents the data acquisition card, 5 denotes the bearing and bearing seat, and 6 corresponds to the magnetic powder brake. Additionally, Figure 4 displays a comparison between the normal intact state bearing and the defective bearing. The red boxes (in the Figure 4) are used to highlight the different types of defects.

In the experiment, the motor speed was set to remain at 500 rpm with no load applied. The sampling rate for sound acquisition was set at 48 KHz.

In this experiment, we collected sound signals of four groups of transmission systems: normal intact state bearing, the bearing of inner ring defect, the bearing of outer ring defect, and the bearing of mixed outer and inner defects. Sound signals were collected in a quiet laboratory environment, with sound signal collection times of 140 s.

**Figure 5:**
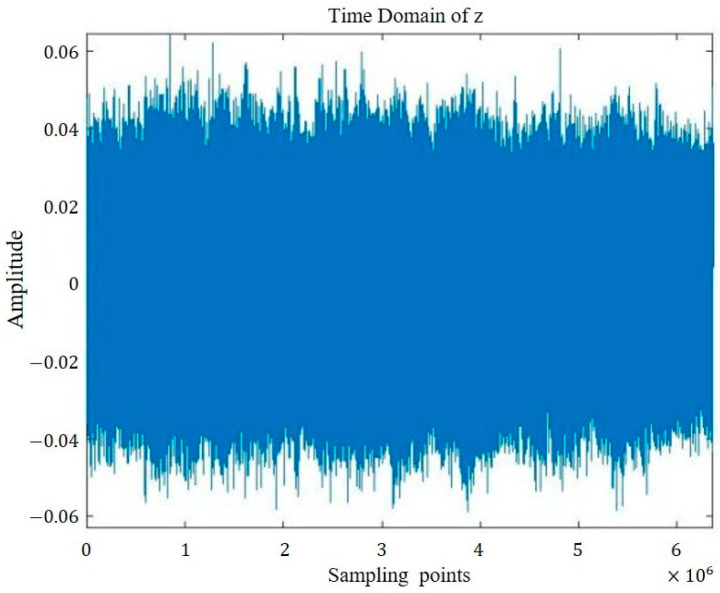
Sound signal of non-defective intact bearing.

**Figure 6:**
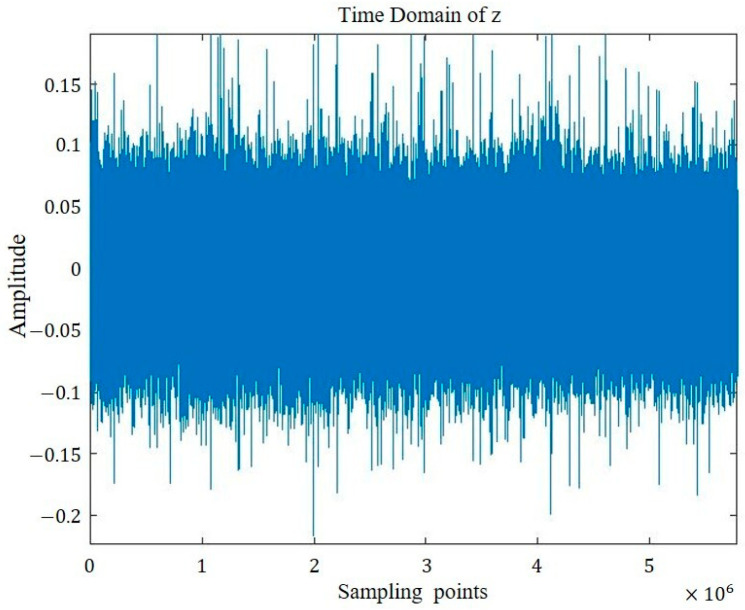
Sound signal of outer ring defect bearing.

**Figure 7:**
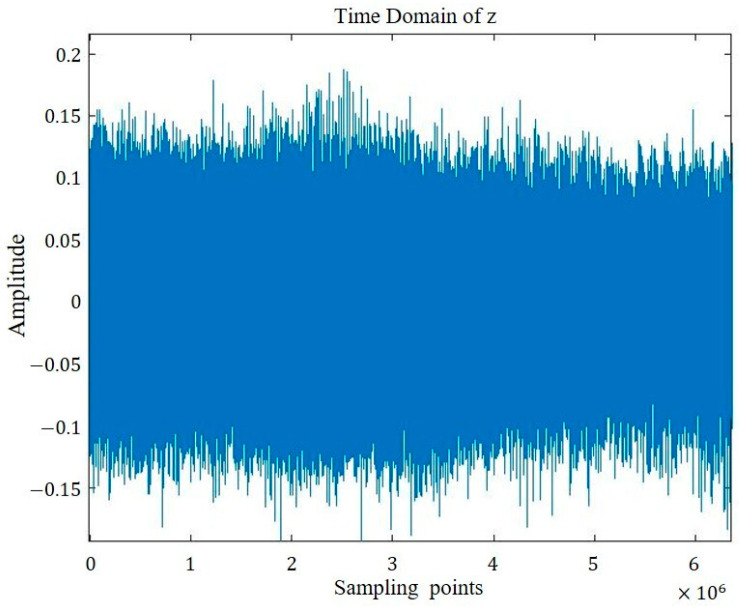
Sound signal of inner ring defect bearing.

**Figure 8:**
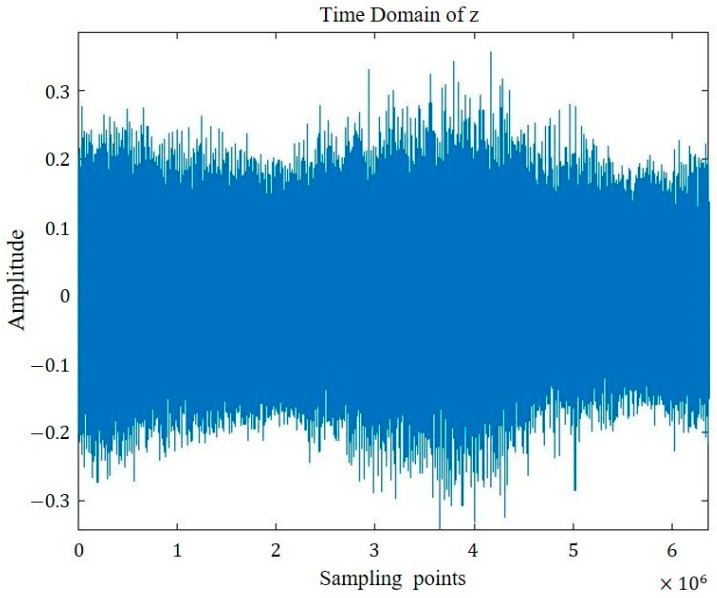
Sound signal of bearing with mixed internal and external defects.

The time domain representations of the four groups of signals are visualized in Figure 5, Figure 6, Figure 7 and Figure 8.

To implement subsequent work effectively with the collected periodic signal, it is essential to divide the entire signal into several segments. Considering that the motor speed is 500 rpm, equivalent to approximately 8.3 revolutions per second and 1 revolution lasting about 0.12 s post-conversion, it can be deduced that 1 revolution corresponds to 5760 sampling points at the sampling frequency of 48 KHz. Maintaining at least 1 revolution in a signal segment is crucial. Hence, when trimming the signal, the segment length was fixed at 7000 with an overlap length of 3500. Upon signal trimming, subsequent feature extraction can be executed.

According to Section 2.1, the corresponding time domain, frequency domain features, and acoustic features of the cropped signal were extracted and further composed into the datasets required in this paper.

### 3.2. Datasets Construction

Through Section 3.1, the corresponding time domain, frequency domain, and acoustic features were extracted and compiled into the datasets essential for the experiment. Three datasets were created: one for time domain and frequency domain features, one for acoustic features, and one for combined complete features. The number of categories and features within each dataset is documented in Table 2, Table 3 and Table 4. In Table 2, Table 3 and Table 4, the numerical values under the columns “Number of 0”, “Number of 1”, “Number of 2”, and “Number of 3” indicate the sample count for each corresponding fault category (i.e., 1800 samples per category). The value in the “Number of Features” column represents the number of features in the dataset (i.e., 22, 39, and 61 features for the three datasets, respectively).These categories include 0 for the normal intact state bearing (nor), 1 for the bearing with outer ring defect (out), 2 for the bearing with inner ring defect (in), and 3 for the bearing with mixed inner and outer ring defects (all2).

After obtaining the datasets, we began to build the algorithm model required for this experiment.

## 4. Fault Diagnosis of Motor Bearing Transmission System Based on CNN–Attention Mechanism–LSTM Model

### 4.1. Construction of Algorithm Model

To perform fault diagnosis of the motor bearing transmission system, a suitable algorithm model is designed based on the characteristics and quantity of the feature datasets. The one-dimensional features extracted guide the selection of an algorithm model that is tailored to such features. Thus, the choice of algorithm model is crucial for effectively achieving the task at hand.

Based on the achievements of acoustic signals in transformer fault diagnosis, it is decided to propose a CNN–attention mechanism–LSTM network model to realize the fault diagnosis of motor bearing transmission system.

The overall framework of the algorithm model can be divided into four parts.

First, the model starts with a one-dimensional convolutional layer aimed at capturing local features in the input data. This is followed by the utilization of a ReLU activation function, enhancing nonlinearity in the module. Subsequently, a maximum pooling layer is employed to reduce feature dimensionality while retaining essential features within the data. Notably, the convolutional layer in this module uses a kernel size of 3 and a stride of 1, while the maximum pooling layer employs a kernel size of 2 with a stride of 2.

The attention mechanism module comes second in the model. Various types of attention mechanisms are available and, for this study, we employ five specific attention mechanism modules, namely SE, CBAM, ECA, SA, and SpatialAttention [43]. These modules selectively enhance the features processed by the one-dimensional convolutional neural network module.

The third part consists of the long short-term memory (LSTM) module, configured with a single layer containing a hidden size of 128.

The fourth part involves a fully connected layer that converts the processed features into output categories.

The construction of the entire algorithm model begins with capturing local features in the input using one-dimensional convolution, followed by enhancing nonlinearity through the ReLU activation function. Subsequently, the feature dimension is reduced via maximum pooling to retain key features. The processed features are then fed into the attention mechanism module for selective enhancement. Next, the features are flattened, a new dimension is added, and the data are then passed to the LSTM module. Finally, the data undergo direct conversion into the output of each category through a fully connected layer.

**Rationale for Hyperparameter Selection**

**Convolutional layer (kernel size = 3, stride = 1):** The kernel size of 3 is a standard choice for 1D-CNNs processing sequential features. It is large enough to capture local patterns and interactions between adjacent feature points, yet small enough to maintain a high degree of model efficiency and avoid overfitting. A stride of 1 is employed to ensure dense, sliding-window feature extraction, preserving the maximum amount of information from the input sequence.

**Maximum pooling layer (kernel size = 2, stride = 2):** It effectively reduces the spatial dimensions (length) of the features by half, thereby decreasing computational complexity and providing a degree of translational invariance. It helps to retain the most salient features while controlling overfitting. We choose maximum pooling over average pooling because bearing fault features are often manifested as impulsive components in the signal/feature domain, and maximum pooling is more effective at preserving such high amplitude, salient activations.

**LSTM hidden size (128):** The hidden state dimension of 128 represents a balance between model capacity and computational efficiency. It provides sufficient representational power to learn complex temporal dynamics from the condensed features output by the preceding CNN and attention modules.

### 4.2. Equipment Environment Configuration

The computer hardware environment parameters and computer software environment parameters used in this paper are shown in Table 5 and Table 6.

### 4.3. Preliminary Results and Analysis

After the preliminary work is completed, training can be carried out using the feature datasets obtained as the input for the constructed algorithm model. The training set, validation set, and test set are divided at a ratio of 8:1:1. The training parameters include a batch size of 128, 200 epochs, a fixed learning rate of 0.001, utilization of the Adam optimizer, and the cross-entropy function as the loss function. Training is conducted using GPU acceleration.

First, the time domain and frequency domain feature dataset is used as input for training. The results are shown in Table 7.

Secondly, the acoustic feature dataset is used as input for training. The results are shown in Table 8.

Finally, the complete dataset consisting of time domain and frequency domain features and acoustic features is used as input for training. The results are shown in Table 9.

Upon analysis of the results, it is evident that the fault diagnosis task is successfully completed, with a good classification effect achieved by utilizing acoustic features. Nonetheless, the accuracy obtained from solely employing time domain, frequency domain features, or a combined feature dataset is significantly lower and falls short of the desired outcome. Subsequent experimentation with these feature groups reveals persistently unstable accuracy levels ranging from 25% to 49%, rendering classification unattainable. Hence, it can be inferred that selecting acoustic features can a achieve classification task.

Next, we explore whether fault diagnosis can be achieved with fewer features. To investigate, we will select and verify the better features.

### 4.4. Feature Selection

Although the features of the datasets are rich, the contribution of different features to classification varies. Therefore, we conducted feature selection. Feature selection can identify and retain the most discriminative features, thereby achieving efficient and robust diagnosis.

Feature selection is a critical process in machine learning and data analysis since it involves choosing a subset of features from the original data that contribute to the model’s predictive performance [4,44]. ReliefF is a feature selection method that quantifies this “discriminative ability” by calculating a weight score for each feature. The higher the weight, the greater the contribution and importance of the feature to classification. The ReliefF algorithm was chosen for its effectiveness in handling multi-class problems and its ability to evaluate features based on their ability to distinguish between instances of different classes that are close to each other [45].

In this paper, we utilized the ReliefF feature selection algorithm [46] to select 18 features and created new datasets for analysis. The feature selection process was carried out twice. Initially, the top nine features were chosen from the time domain and frequency domain feature dataset and combined with the top nine features from the acoustic feature dataset, resulting in a 9 + 9 integrated dataset. Subsequently, the top 18 features from the acoustic feature set were selected to form the selected acoustic feature dataset. Given the large number of features involved, we showed the importance scores and rankings of the top 9 time domain and frequency domain features, as well as the top 18 acoustic features. To mitigate the impact of randomness, each set of experiments was repeated four times, and the features that appeared most frequently were selected for analysis. Considering the length of this paper, only one specific result of the selection will be presented here. Table 10 and Table 11 present the results from one experiment conducted. The final features selected for the two new datasets are detailed in Table 12 and Table 13, while the specific descriptions of the two new datasets are provided in Table 14 and Table 15.

### 4.5. Results and Analysis After Feature Selection

The feature datasets obtained after feature selection are used as input for training. Other parameters remain unchanged as above. The results are shown in Table 16 and Table 17:

From the above two sets of tables, it is evident that classification cannot be achieved when using the complete selection feature set that combines the two. Conversely, when utilizing the selected acoustic feature set, the classification performance is very good. Therefore, it can be concluded that the acoustic features chosen by the feature selection algorithm are effective in achieving classification.

To shed light on the advantages of selecting acoustic features for fault diagnosis, two parameters—parameter quantity and estimated total size—are introduced as reference points for comparison. The results of this analysis are presented in Table 18. This approach allows us to examine the efficiency and effectiveness of selecting acoustic features both before and after performing fault diagnosis tasks.

**Figure 9:**
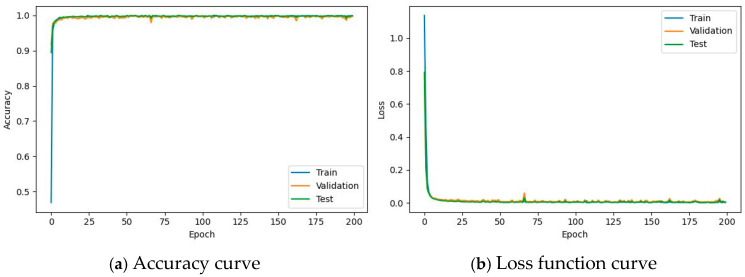
Accuracy curve and loss function curve of CNN-ECA-LSTM algorithm model.

It is evident from Table 18 above that the ReliefF feature selection algorithm significantly reduces the number of parameters and estimated total size while maintaining the accuracy almost unchanged. This indicates the effectiveness of feature selection for acoustic features in fault diagnosis of motor efficiently bearing transmission systems. Consequently, fewer acoustic features can be utilized to achieve this task. After a comprehensive comparison of the above five models, the CNN-ECA-LSTM algorithm model was finally selected as the final structural choice for this task. The ECA attention mechanism module consists of an adaptive average pooling layer (AdaptiveAvgPool1d), a one-dimensional convolutional layer (Conv1d), and a sigmoid activation function, which enhances the features of important channels and suppresses the features of unimportant channels in a lightweight manner. The accuracy curve and loss function curve of its training are shown in Figure 9.

**Figure 10:**
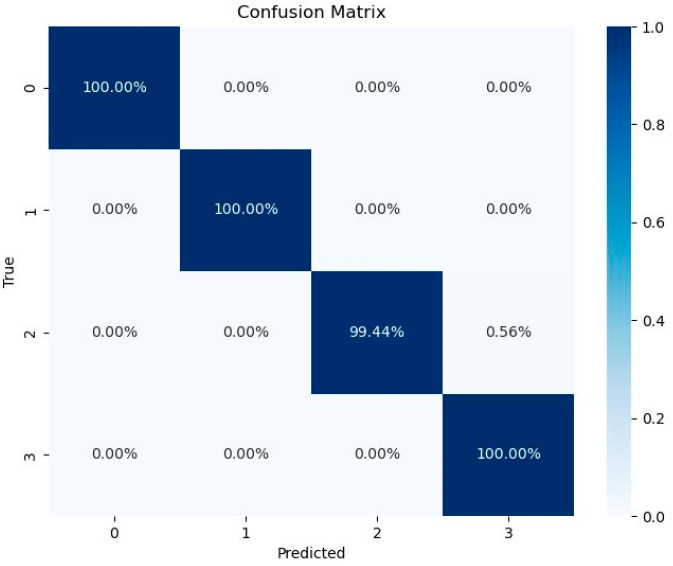
Confusion matrix of CNN-ECA-LSTM algorithm model.

The confusion matrix is shown in Figure 10. These categories include 0 for the normal intact state bearing (nor), 1 for the bearing with outer ring defect (out), 2 for the bearing with inner ring defect (in), and 3 for the bearing with mixed inner and outer ring defects (all2).

**Figure 11:**
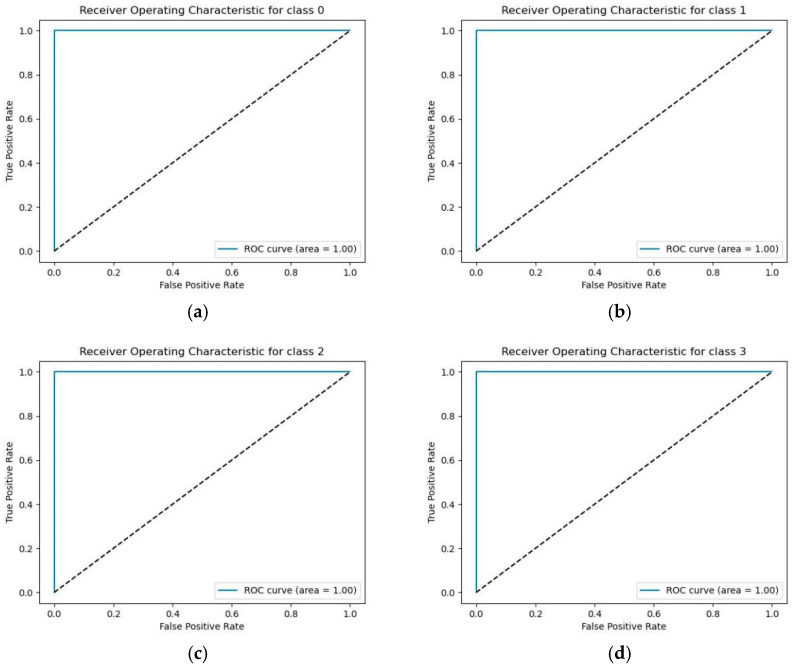
ROC curves and corresponding AUC values for each classification (from 0 to 3 from left to right are (**a**) Receiver Operating Characteristic for class 0, (**b**) Receiver Operating Characteristic for class 1, (**c**) Receiver Operating Characteristic for class 2, (**d**) Receiver Operating Characteristic for class 3).

To ensure experimental reliability, we used the ROC curve and corresponding AUC value as references. In a multi-classification task, we examined the ROC curve and AUC value for each class. These results are displayed in Figure 11.

The dashed line represents the performance benchmark for a random classifier. When used as a reference, if a model’s ROC curve lies above this line, it indicates that the model possesses effective discriminatory power. The AUC value of each classification is 1, which means that the model can perfectly distinguish between positive and negative examples, that is, the performance of the model is optimal. The precision, recall and F1 score are shown in Table 19.

Finally, we repeated the experiments on the selected algorithm model several times, and the results are shown in Table 20.

The average of the seven results is 99.90% and the average training time is 36.64 s.

Our entire process involves only signal cropping without any additional signal processing step from the perspective of signal processing. We are able to achieve the recognition task with an average accuracy of 99.90%. Additionally, the AUC value corresponding to the ROC curve of each classification is 1, demonstrating the effectiveness of our method. Realize the classification of fault types in bearings and research on bearing fault diagnosis methods using sound signals and one-dimensional acoustic features.

## 5. Conclusions

This paper mainly conducts bearing fault diagnosis by collecting sound signals and extracting corresponding acoustic features (MFCC). This research proposes a fault diagnosis method for the bearing based on acoustic features and deep learning. Initially, sound signals from four types of bearings—the normal state bearing, the bearing of inner ring defect, the bearing of outer ring defect, and the bearing of mixed inner and outer defects—are gathered, and the respective acoustic features are extracted. Subsequently, the one-dimensional acoustic features are input into the CNN–attention mechanism–LSTM model for fault type classification. To optimize the model, a feature selection algorithm is employed to streamline the features, resulting in a reduced number of algorithm model parameters and estimated total size without compromising accuracy. Ultimately, the CNN-ECA-LSTM algorithm model is chosen based on its superior performance.

The acoustic diagnosis model proposed in this study demonstrates significant effectiveness in experimental environments; however, its feasibility for practical deployment is impacted by the following key challenges: firstly, ambient noise can diminish the model’s robustness; secondly, training under fixed operating conditions limits its generalization capability in variable load and speed scenarios. In order to develop a comprehensive and practical bearing fault diagnosis method based on sound signals, future research must focus on improving the adaptability of the model in complex industrial environments and expanding the range of fault types it can diagnose.

## Figures and Tables

**Table 1:** The names and abbreviations of the extracted features.

Feature	Abbreviation	Feature	Abbreviation	Feature	Abbreviation	Feature	Abbreviation
maximum	max	standard deviation	std	peak factor	pf	frequency standard deviation	fsd
minimum	min	kurtosis	kurtosis	pulse factor	pf1		
average	mean	skewness	skewness	clearance factor	cf		
median	median	root mean square	rms	center of gravity frequency	cgf		
peak to peak value	pk	mean squared value	msv	mean square frequency	msf		
rectification average	rv	root square amplitude	rsa	root mean square frequency	rmsf		
variance	var	waveform factor	wf	frequency variance	fv		

**Table 2:** Time domain and frequency domain feature dataset.

Category	Number of 0	Number of 1	Number of 2	Number of 3	Number of Features
Time domain and frequency domain feature	1800	1800	1800	1800	22

**Table 3:** Acoustic feature dataset.

Category	Number of 0	Number of 1	Number of 2	Number of 3	Number of Features
Acoustic feature	1800	1800	1800	1800	39

**Table 4:** A complete feature dataset consisting of two combinations.

Category	Number of 0	Number of 1	Number of 2	Number of 3	Number of Features
Complete feature	1800	1800	1800	1800	61

**Table 5:** Computer hardware environment parameters.

Hardware	Parameter Configuration
Cpu	AMD Ryzen 7 5800H with Radeon Graphics 3.20 GHz
Graphics card	NVIDIA GeForce RTX 3060 Laptop GPU
Memory	16.0 GB

**Table 6:** Computer software environment parameters.

Software	Configuration
Operating system	Windows 11
Development platform	MATLAB R2023a and PyCharm Community Edition 2022.3.2
Development framework	Pytorch2.1

**Table 7:** Results of time domain and frequency domain feature dataset.

Attention Mechanism Module	Test Set Accuracy (%)	Training Time (s)
SE	27.22	38.40
CBAM	48.75	53.32
SpatialAttention	47.50	37.61
SA	49.31	44.28
ECA	43.19	35.78

**Table 8:** Results of acoustic feature dataset.

Attention Mechanism Module	Test Set Accuracy (%)	Training Time (s)
SE	99.86	38.36
CBAM	99.86	50.50
SpatialAttention	99.86	37.47
SA	99.86	43.70
ECA	100.00	36.82

**Table 9:** Results of complete dataset.

Attention Mechanism Module	Test Set Accuracy (%)	Training Time (s)
SE	47.92	42.80
CBAM	45.28	48.82
SpatialAttention	25.00	37.75
SA	44.44	47.69
ECA	25.00	36.12

**Table 10:** Importance score and ranking of time domain and frequency domain features’ selection.

Select	Features	ReliefF
1	wf	0.0504
2	kurtosis	0.0444
3	fsd	0.0381
4	fv	0.0364
5	cf	0.0288
6	rsa	0.0279
7	pf1	0.0195
8	skewness	0.0169
9	rv	0.0165

**Table 11:** Importance score and ranking of the top 18 acoustic features’ selection.

Select	Features	ReliefF	Select	Features	ReliefF
1	mfcc_2	0.074	10	mfcc_6	0.012
2	mfcc_10	0.0479	11	mfcc_delta_4	0.0104
3	mfcc_5	0.0403	12	mfcc_delta_delta_3	0.0091
4	mfcc_1	0.04	13	mfcc_delta_5	0.0091
5	mfcc_11	0.0383	14	mfcc_delta_7	0.0078
6	mfcc_4	0.0321	15	mfcc_delta_3	0.007
7	mfcc_9	0.0233	16	mfcc_delta_2	0.0069
8	mfcc_3	0.0189	17	mfcc_delta_6	0.0068
9	mfcc_7	0.0136	18	mfcc_delta_delta_7	0.0062

**Table 12:** Final selection of the first nine time domain and frequency domain features.

Category	Specific Expression
Time domain and frequency domain features	wf; kurtosis; fsd; fv; cf; rsa; pf1; skewness; rv

**Table 13:** Final selection of the top 18 acoustic features.

Category	Specific Expression
Acoustic features	mfcc_2; mfcc_10; mfcc_1; mfcc_11; mfcc_5; mfcc_4; mfcc_9; mfcc_3; mfcc_6; mfcc_7; mfcc_delta_7; mfcc_delta_delta_3; mfcc_delta_4; mfcc_delta_6; mfcc_delta_5; mfcc_delta_2; mfcc_delta_3; mfcc_delta_11

**Table 14:** Acoustic feature dataset after feature selection.

Category	Number of 0	Number of 1	Number of 2	Number of 3	Number of Features
Acoustic feature	1800	1800	1800	1800	18

**Table 15:** Integrated feature dataset after feature selection.

Category	Number of 0	Number of 1	Number of 2	Number of 3	Number of Features
Integrated feature	1800	1800	1800	1800	9 + 9

**Table 16:** Results of integrated feature dataset after feature selection.

Attention Mechanism Module	Test Set Accuracy (%)	Training Time (s)
SE	25.00	41.14
CBAM	25.00	50.45
SpatialAttention	25.00	37.50
SA	25.00	44.59
ECA	25.00	35.96

**Table 17:** Results of acoustic feature dataset after feature selection.

Attention Mechanism Module	Test Set Accuracy (%)	Training Time (s)
SE	100.00	38.90
CBAM	99.86	50.19
SpatialAttention	99.86	37.74
SA	99.72	44.55
ECA	99.86	36.31

**Table 18:** Comparison of acoustic feature dataset results before and after feature selection.

Attention Mechanism Module	Test Set Accuracy (%)	Training Time (s)	Parameter Quantity	Estimated Total Size (MB)	Test Set Accuracy After Selection (%)	Training Time After Selection (s)	Parameter Quantity After Selection	Estimated Total Size After Selection (MB)
SE	99.86	38.36	214,628	0.86	100.00	38.90	132,708	0.53
CBAM	99.86	50.50	214,642	0.86	99.86	50.19	132,722	0.53
SpatialAttention	99.86	37.47	214,602	0.86	99.86	37.74	132,682	0.53
SA	99.86	43.70	214,602	0.87	99.72	44.55	132,682	0.53
ECA	100.00	36.82	214,644	0.86	99.86	36.31	132,724	0.53

**Table 19:** Results of precision, recall and F1 score.

Precision	Recall	F1 Score
0.9986	0.9986	0.9986

**Table 20:** Results of CNN-ECA-LSTM.

Number of Experiments	Test Set Accuracy (%)	Training Time (s)
1 (The above experiment)	99.86	36.31
2	100.00	36.74
3	100.00	36.95
4	99.86	36.57
5	100.00	36.23
6	99.72	36.52
7	99.86	37.15

## Data Availability

The data cannot be made publicly available upon publication because no suitable repository exists for hosting data in this field of study. The data that support the findings of this study are available upon reasonable request from the authors.
